# The Moderator Role Of Gender In The Relationship Between Behavioral Inhibition And Parental Behaviour In Preschool Children

**DOI:** 10.1192/j.eurpsy.2022.2211

**Published:** 2022-09-01

**Authors:** R.S. Sulyok, M. Miklósi

**Affiliations:** 1 Eötvös Loránd University Faculty of Education and Psychology, Department Of Developmental And Clinical Child Psychology, Budapest, Hungary; 2 Eötvös Loránd University, Department Of Developmental And Clinical Child Psychology, Budapest, Hungary

**Keywords:** gender, anxiety disorder, Behavioral inhibition, parental behavior

## Abstract

**Introduction:**

Preschool Behavioural Inhibition (BI) was found to be a temperamental risk factor of anxiety disorders in later life; especially in women. Similarly, previous research revealed that parental behaviour plays a major role in the development and maintenance of anxiety disorders. Gender differences in parental responses to child’s temperament may contribute to the stronger association between BI and anxiety disorders in females.

**Objectives:**

We aimed to examine the moderating effect of child’s gender in the association between child’s BI and parenting behaviour in a non-clinical sample of parents of preschool children.

**Methods:**

A cross-sectional sample of parents (N=94) of preschool children (girls 47.4%) filled out the Behavioural Inhibition Questionnaire (BIQ) and the Multidimensional Assessment of Parenting Scale (MAPS).

**Results:**

Child’s gender was found to moderate the relationships between BIQ scores and MAPS Supportive Parenting (F(3,90)=4.350, p=.007, R2=.127), as well as Hostile Parenting (F(3,89)=3.478, p=.019, R2=.105). In boys, higher BIQ scores were associated with higher levels of Supporting Parenting (b=.005, p=.027), while in girls this association was reversed (b=-.004, p=.037). Furthermore, in boys, no association was found between BIQ scores and Hostile Parenting (b=.005, p=.835); however, higher BIQ scores were related to higher levels of Hostile Parenting in girls (b=.067, p<.001).

**Conclusions:**

Our results suggest that parental responses to their preschool child’s Behavioural Inhibition may vary as a function of child’s gender. This may lead to gender differences in developmental pathways to anxiety disorders.

**Disclosure:**

No significant relationships.

